# The Evaluation of Oxidative Stress Parameters in Serum Patients with Relapsing-Remitting Multiple Sclerosis Treated with II-Line Immunomodulatory Therapy

**DOI:** 10.1155/2017/9625806

**Published:** 2017-09-12

**Authors:** Bożena Adamczyk, Sławomir Wawrzyniak, Sławomir Kasperczyk, Monika Adamczyk-Sowa

**Affiliations:** ^1^Department of Neurology in Zabrze, School of Medicine with the Division of Dentistry in Zabrze, Medical University of Silesia, Katowice, ul. 3 Maja 13-15, 41-800 Zabrze, Poland; ^2^Department of Neurology, 10th Military Research Hospital and Polyclinic, Independent Public Healthcare Centre, ul. Powstańców Warszawy 5, 85-681 Bydgoszcz, Poland; ^3^Department of Biochemistry in Zabrze, School of Medicine with the Division of Dentistry in Zabrze, Medical University of Silesia, Katowice, ul. 3 Jordana 19, 41-808 Zabrze, Poland

## Abstract

**Objectives:**

The assessment of oxidative stress (OS) in serum relapsing-remitting multiple sclerosis patients treated with II-line immunomodulatory therapy (fingolimod, natalizumab) compared to newly diagnosed patients (de novo group) treated with interferon (IFN) beta and controls. The relationship between OS parameters and gender, age, disease duration, Expanded Disability Status Scale, annualized relapse rate, MRI lesions in patients treated with II-line.

**Materials and Methods:**

One hundred and twenty-one patients with RRMS were enrolled in the study. Patients were divided into groups: de novo group, IFN, fingolimod (FG), natalizumab (NT), and controls. Lipid hydroperoxides (LHP), malondialdehyde (MDA), lipofuscin (LPS), and total oxidative status (TOS) were determined.

**Results:**

LHP, MDA, and TOS were lower in NT and FG groups compared to the de novo group. Levels of OS were different between NT and FG patients and the IFN group. Women treated with FG and NT had lower MDA, LPH, and TOS than women who were not treated while in men only LPH was lowered. Positive correlations were found between MDA, LHP, TOS, and ARR in the NT group.

**Conclusion:**

The II-line immunomodulatory treatment decreased OS particularly among women. No difference in OS levels was observed between II-line therapy and IFN beta.

## 1. Introduction

Multiple sclerosis (MS) is a multifactorial disease of the central nervous system (CNS) characterized by inflammation and demyelination [1]. It is connected with neuroinflammation, demyelination, and axonal loss [1]. The etiopathology remains unclear. Genetic and environmental factors are suspected to play a role in the pathogenesis, and recently, attention has been also paid to oxidative stress (OS) as one of the main factors responsible for demyelination [2–4]. The imbalance between OS agents and antioxidants leads to OS activating the inflammatory process [5, 6].

Hyperactivity OS enzyme is responsible for the production of free radicals [7, 8]. These, in turn, attack the various classes of biomolecules (proteins, lipids, carbohydrates, DNA, and RNA), leading to the damage at the level of mitochondria and ion channels, which activates apoptotic pathways [9, 10]. In addition, the energy balance of neurons is disturbed and consequently contributes to neurodegeneration [11–13]. Therefore, understanding the relationship between OS and the course of multiple sclerosis is so crucial.

Multiple sclerosis may occur in several forms, that is, relapsing-remitting (RRMS), secondary progressive (SPMS), and primary progressive (PPMS). Currently, MS treatment is based on immunomodulatory therapy. The I-line of treatment includes interferon (IFN) beta, glatiramer acetate, dimethyl fumarate, and teriflunomide, whereas the II-line includes fingolimod (FG), natalizumab (NT), and alemtuzumab.

The most widely used I-line drugs are interferon (IFN) beta and glatiramer acetate. Their effectiveness is comparable in the treatment of the RRMS [14]. The more aggressive forms of RRMS are treated with II-line drugs, mainly FG and NT.

Due to the fact that the effectiveness of therapy is still limited, new therapeutic strategies are constantly under investigation [15, 16]. It seems that the beneficial effect on the inflammatory processes is insufficient. The participation of redox processes in the pathogenesis of MS has been recently highlighted [17]. The parameters of OS are investigated for their impact on the course of the disease. Biomarkers of OS may be used for the assessment of the prognosis of exacerbation or the treatment response [18].

It is believed that new immunomodulatory drugs may have an influence on OS level in patients with MS [18]. In particular, NT and FG are poorly understood in this respect. The study on OS biomarkers in the immunomodulatory therapy has been an important element of further research on MS. This is a new issue that has not been explored yet.

## 2. Objectives

We assessed OS parameters in the blood serum of RRMS patients in the Clinic of Neurology in Zabrze, Poland. The parameters were assessed in patients treated with II-line immunomodulatory therapy compared to newly diagnosed RRMS patients (de novo group), RRMS patients treated with IFN beta, and healthy subjects. We assessed the relationship between OS parameters and gender, age, disease duration, degree of disability in the Expanded Disability Status Scale (EDSS), ARR, and MRI Gd + lesions.

## 3. Materials and Methods

One hundred and twenty-one patients diagnosed with RRMS according to the McDonald criteria of 2010 were enrolled in the study. In addition, the study involved 41 volunteers (controls) that did not suffer from MS.

The inclusion criteria (study group) are the following:

- RRMS patients diagnosed according to the 2010 McDonald criteria. The immunomodulatory treatment with interferon *β*-1a, interferon *β*-1b, glatiramer acetate, fingolimod, and natalizumab
- 10 ml of venous blood
- Age ≥ 18 years
- Female or male
- Written informed consent for participation in the study
- Patient free from relapse
- Blood samples were not taken immediately after drug administration.

The exclusion criterion (study group) is the following:

- Lack of consent to participate in the study.

The inclusion criteria (control group) are the following:

- Generally healthy people (diagnosis of MS was excluded; treatment of hypertension was not a contraindication)
- Nonsmokers.

The exclusion criteria (control group) are the following:

- Smokers
- Lack of consent to participate in the study.

Patients were divided into the following groups, according to the type of immunomodulatory therapy:

1. De novo group—patients newly diagnosed with RRMS without immunomodulatory therapy (24 patients)
2. IFN—RRMS patients treated with IFN beta-1a (30 *μ*g i.m. weekly or 44 ug s.c. 3 times per week) or interferon beta-1b (250 *μ*g every second day s.c.) (32 patients)
3. FG—RRMS patients treated with FG (0.5 mg/daily p.o.) (39 patients)
4. NT—RRMS patients treated with NT (300 mg once a month IV) (26 patients)
5. Control—a control group of nonsmoking healthy volunteers (41 persons).

A sample of 10 ml venous blood were obtained and placed in chilled tubes with 1 mg/ml EDTA-K3 as anticoagulant. After centrifugation of blood samples, the obtained serum was frozen at −80°C. The following OS parameters were determined:

- concentration of lipid hydroperoxides (LHP),
- malondialdehyde (MDA),
- lipofuscin (LPS),
- total oxidative status (TOS).

The determination of the level of the parameters was conducted using standard methods.

The concentration of LHP in serum was determined by the method of Sodergren et al. Xylene xangan was used. The procedure was based on the oxidation of iron (II) ions to iron (III) ions in acidic medium. Then, iron (III) ions with xylene orange form a colorful complex, up to a blue-purple coloration. The reading was made with a 560 nm filter using the Perkin Elmer VICTOR-X3 reader. The concentration was read from the calibration curve prepared with the aid of appropriate H_2_O_2_ concentrations. Values are expressed in *μ*mol/l [19].

The MDA concentration was determined in serum using the MDA reaction with thiobarbituric acid according to Ohkawa. For reading, the LS45 spectrometer from Perkin Elmer was used at 515 nm (absorbance) and 552 nm (emission) spectrophotometer. Spectrofluorometric reading, unlike spectrophotometry (at 532 nm), is more specific, and it does not interfere with hemoglobin; no bile duct interference is observed. The method was modified by adding sodium sulphate and BHT, which further increased the specificity of the method. MDA concentrations were read from the standard curve using standard 1,1,3,3-tetraethoxypropane and expressed in micromoles per liter of serum (*μ*mol/l) [20].

Serum LPS concentration was determined according to the method of Tsuchida et al. Ethanol-ether mixtures 3 : 1 (*v*/*v*) were added to the serum, shaken, and centrifuged. The fluorescence intensity was determined using a Perkin Elmer LS45 spectrophotometer at 345 nm (absorbance) and 430 nm (emission) wavelengths in a dissolved solid.

The values are expressed in relative lipid extract fluorescence (RF), where the value of 100 RF corresponds to the fluorescence of the solution of 0.1 *μ*g/ml quinidine sulphate in 0.1 N sulfuric acid. LPS concentrations are shown in RF [21].

The TOS assay is based on the oxidation of iron (II) ions to iron (III) ions in acidic medium. Then, iron (III) ions with xylene orange form a colorful complex, up to a blue-purple coloration.

The absorbance readings were made with a 560 nm filter on the PerkinElmer VICTOR-X3.

The concentration was calculated from the calibration curve using H_2_O_2_ as the standard.

Values are expressed in *μ*mol/l [22].

Demographic data, clinical disease onset, disease duration, clinical form of MS, the type of treatment, ARR, the degree of disability in the EDSS scale, and lesions on MRI were obtained from medical databases and the Department of Neurology. The results were stored in the database prepared specifically for this purpose in Microsoft Excel.

STATISTICA 9.1 was used for the statistical analysis. *p* < 0.05 was considered statistically significant. Variables were estimated for the normality of the distribution by the Shapiro-Wilk test. Demographic characteristics and all the results were expressed as the number (*N*), arithmetic mean, standard deviation, median, interquartile range, and percentage (%).

Homogeneity of continuous variables between groups was analyzed using the parametric ANOVA test (for normally distributed variables) or the nonparametric test Kruskal-Wallis ANOVA (for variables whose distribution was not normal).

Post hoc analysis using Tukey's test with the Bonferroni correction was conducted in the case of statistically significant differences. The Student *t*-test and the nonparametric Mann–Whitney *U* test were used to compare the two groups. The homogeneity of the study groups in terms of qualitative variables was analyzed by Pearson chi-square test. The numbers were compared between groups using multiway tables and the chi-square test. The relationship between attributes was evaluated by the linear Pearson correlation.

The study was approved by the Bioethics Committee of the Medical University of Silesia in Katowice, Poland. This study was conducted in accordance with the Helsinki criteria for patient trials. The approval number of The Bioethical Commission was KNW/0022/KB1/37/16 of 19th April 2016.

## 4. Results

A group of patients with RRMS and the control group proved to be homogeneous in terms of gender and age. Women accounted for 66.94% of patients and 78.05% of the control (Table 1).

Table 2 presents a detailed characteristics of the study groups, considering the clinical and radiological indicators assessing the degree of disease activity such as disease duration, EDSS, ARR, Gd + MRI lesions, and T2 MRI lesions. Study groups did not differ from these data. The newly diagnosed patients (de novo group) were the oldest group, and the NT group was the youngest. The number of female patients was higher compared to the male patients (Table 2).

Table 3 shows the analysis of FG and NT groups before and during the II-line treatment. Some patients from the FG group (35.90%) had been previously treated with IFN 1a, 46.15% with IFN 1b and 17.95% with glatiramer acetate whereas in the NT group - 40%, 45% and 50%, respectively. Table 3 also presents time of I-line treatment (years) and the comparison of the study groups, considering clinical and radiological indicators assessing the degree of disease activity before treatment of II-line (EDSS, ARR, Gd + MRI lesions, T2 MRI lesions). The time of I-line treatment for the FG group was 2 ± 2 years and for the NT group 2 ± 2.5 years, and the time of II-line treatment 2.14 ± 1.39 and 2.09 ± 1.27 years, respectively.

The treatment effects are presented as the percentage of patients free from relapse (FG group 71.43% versus NT group 87.5%), free of clinical disease progression at the end of the observation (FG group 28.57% versus NT group 33.33%), and radiological disease progression at the end of the observation (FG group 93.75% versus NT group 95.83%). The number of T2 MRI lesions was represented as N number (FG group 20.25 ± 1.86 versus NT group 18.50 ± 4.90). The results did not differ significantly between the two groups (Table 3).

### 4.1. The Analysis of Selected OS Parameters in Groups According to the Type of Treatment (Table 4)

Patients with RRMS were divided into four groups. The fifth group was the control group.

All groups were compared with respect to the selected parameters of OS in serum. The study groups showed the difference in the concentrations of LHP, MDA, LPS and TOS. The post hoc analysis was conducted for FG and NT groups only for statistically significant *P*-value.

Significantly lower concentrations of LHP, MDA and TOS were observed in NT and FG groups compared to the de novo group. In addition, a lower concentration of LPS was observed in the NT group compared to the de novo group. Both NT and FG groups were not different from the IFN group in terms of OS parameters. Significantly higher MDA concentrations were noted in the groups treated with II-line immunomodulatory therapy as compared to the control group. The IFN group also had significantly lower all parameters of OS compared to the de novo group, but they were higher in the IFN group compared to the control group. It should be stressed that in the FG and NT groups only the MDA concentration was higher than that in the control. Other parameters were not different from the control group.

### 4.2. The Analysis of the Groups by Gender (Women) (Table 5)

Women were selected from all of the groups. The post hoc analysis revealed that all women treated with II-line drugs had lower levels of OS parameters such as MDA, LPH, and TOS compared to women who had not been previously treated. Women in the IFN group had lower levels of MDA, LHP, and TOS compared to women diagnosed de novo, but MDA and LHP levels were higher in the IFN group compared to healthy women. However, in the FG and NT groups, this difference was not observed.

### 4.3. The Analysis of the Groups by Gender (Men) (Table 6)

Then, men were selected from all of the groups. The post hoc analysis revealed that all the men treated with II-line drugs had a lower level of LHP than men who had not been previously treated. In men, OS parameters from the IFN group were not different from the OS parameters in the de novo group. Healthy men had lower most of these parameters compared to men diagnosed de novo and the IFN group.

### 4.4. The Analysis of the Most Important Correlations for RRMS Patients Treated with II–Line (Table 7)

Moderately positive correlations were found in the NT group between the concentrations of MDA, LHP, TOS, and ARR. No other significant correlation was found.

In the IFN group, only a positive correlation was found between Gd + MRI lesions and LHP and MDA and TOS concentrations.

## 5. Discussion

**Figure 1:**
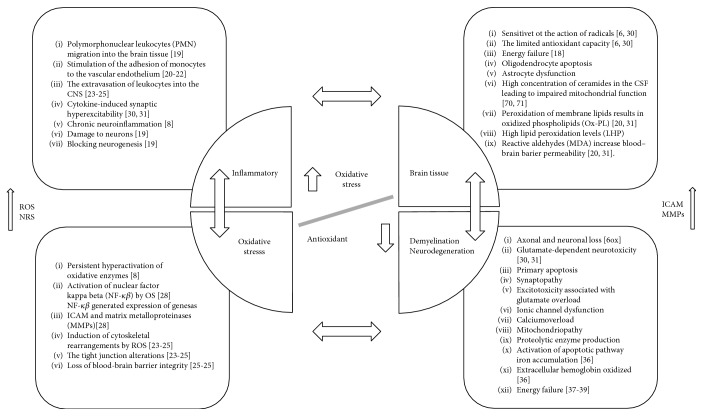
Possible significance of oxidative stress in the pathomechanism of multiple sclerosis. CNS: central nervous system; ROS: reactive oxygen species; RNS: reactive nitrogen species; OS: oxidative stress; ICAM: intercellular adhesion molecule.

The contribution of OS to MS is very complex and linked to many mechanisms (Figure 1). Currently, it is believed that MS is a biphasic disease [2]. Initially, inflammatory processes dominate, and the process is associated with polymorphonuclear leukocyte (PMN) migration into the brain tissue [23] and stimulates the adhesion of monocytes to the vascular endothelium [24–26]. The extravasation of leukocytes into the CNS [27–29] generates cytokine-induced synaptic hyperexcitability [30, 31] and ultimately leads to chronic neuroinflammation [8]. These processes induce OS and initiate inflammatory processes by persistent hyperactivation of oxidative enzymes [8], activation of nuclear factor kappa beta (NF-*κβ*) [32], and loss of the blood-brain barrier integrity [27–29]. It seems that the key element may be an imbalance between oxidative and antioxidative processes in MS patients. This can be expressed through the imbalance between the concentration of compounds such as lipid peroxidation levels (MDA, LHP), carbonyl protein content, DNA damage, GSH, SOD, GST [23], CAT activities, vitamins C and E, nonprotein thiol content [33], and transcription factor Nrf2 which is responsible for important antioxidant pathway [18]. The brain tissue is susceptible to the action of radicals [6, 34] due to the limited antioxidant capacity [6, 34]. Lipid peroxidation results in oxidized phospholipids (Ox-PL) [24, 35], energy failure [18], and consequently oligodendrocyte apoptosis and astrocyte dysfunction. All these processes generate demyelination and neurodegeneration [36–39]. This problem, however, is beyond the scope of the present paper. The main problem is related to the explanation of the relationship between peripheral OS markers and OS in the CNS. Our paper attempted to investigate this problem with a particular regard to II-line (FG, NT) drugs.

This study showed an impact on OS parameters in MS patients treated with II-line immunomodulatory therapy. Better understanding of the effects of II-line drugs may help explain the mechanism of OS in the pathogenesis of MS. It is possible that this direction of research may allow in the future to introduce new therapies based on the oxidative/antioxidant system. What is more, there may be some possibility of using new markers to evaluate treatment response [18].

Unfortunately, there are some limitations to these markers, due to the fact that the level of OS may depend on many factors such as age, gender, activity level, diet, smoking, and exposure to toxic substances. Our study took into account the gender and age of patients with MS and the control group.

Both in NT and FG groups, a reduction was observed in the level of parameters such as TOS, LHP, and MDA compared to the newly diagnosed patients. Similar results were also obtained in the IFN and the control group.

Some study suggested that FG might even change MDA levels in the hippocampus in the rat model of autism [40]. The effect of reducing the MDA level by FG was showed in the other study where its effect on reducing torsion/detorsion induced testicular injury. FG, by activating the S1P receptors, reduced the level of MDA activity, concentration of interleukin-1*β*, and myeloperoxidase activity [41]. The results of these studies could not be interpreted intermittently because the mechanism of action of FG is complex and may depend on the type of tissue and the cells on which the drug was active. Fingolimod modulates the activity of sphingosine-1-phosphate receptors (S1PR1), thereby inhibiting T lymphocyte migration from the central nervous system [42] lymph nodes, and thus acts on the first step of MS—neuroinflammatory. Serdar et al. presented the beneficial effects of FG in neonatal oxygen-induced brain injury and the contribution of FG in the protection of oligodendrocytes. The authors suggest that modulation of peripheral lymphocyte trafficking may be less relevant [43].

Researchers suggested a multidirectional and very complex mechanism of FG action, also listing immunosuppression and antitumor activity [44, 45].

Early studies suggested that FG can induce apotheosis of cells as well as protect cells from OS. One study demonstrated that FG could induce apoptosis of tumor cells, lymphocytes, and atypical neutrophils by rapid translocation of heat shock protein 27 to the cell surface. The authors of that study suggested that FG acted through the necrosome signalling complex and the OS machinery [46]. Similar findings which showed that FG could induce reactive oxygen species (ROS) accumulation are reported in other studies where cells lacking the stress-activated MAP kinase SPC1/Sty1 exhibited higher sensitivity to FG and higher ROS levels [47]. On the other hand, Santos-Gallego et al. hypothesized that activation of the sphingosine-1-phosphate (S1P) receptor with FG inhibited apoptosis [48]. Also FG might have a protective effect on the PC12 cells exposed to hydrogen peroxide [49].

Studies on FG in other diseases showed its positive effect in acute stroke [49, 50], and FG might improve prognosis in intracerebral haemorrhage in rodents [51], which could be beneficial to the CNS.

In our study, lipid peroxidation (LPH) in patients treated with II-line was more severe compared to controls, and in the IFN group all examined parameters were higher than those in the control group. However, the level of the selected OS parameters in patients treated with IFN beta and II-line drugs was not different.

The data on the impact of IFN beta on the reduction of OS are still unclear. It is supposed that IFN beta treatment in combination with other agents or antioxidants could decrease the level of oxidative parameters. For instance, the treatment with IFN beta and glatiramer was reported to have reduced tumor necrosis factor alpha (TNF-*α*); however, it did not affect other ROS/reactive nitrogen species (RNS) [52]. Another study revealed that nitric oxide levels and its reactive derivatives (NOx) were higher in the IFN beta group compared to the FG and NT groups and healthy controls [53].

It is difficult to assess the participation of IFN beta in oxidative processes. The suppression of OS by IFN beta cannot be ruled out as compared to patients treated with II-line therapy.

Patients treated with NT had a lower TOS and lower lipid peroxidation levels than untreated patients. Several authors suggested the impact of the monoclonal antibody on the whole OS level in MS patients [54–56]. Some conducted studies related to a reduction in carbonylated protein levels, myeloperoxidase levels, and myeloperoxidase/neutrophil granulocyte ratio [55, 56]. Generally, studies on the relationship between NT and the lipid peroxidation are not sufficient.

Scientific studies confirmed that certain genotypes of detoxification enzymes such as NQO1 and the GSTP gene possibly showed a better clinical outcome after NT therapy [57]. One of the studies indicated the participation of melatonin in the reduction of OS in patients treated with NT [58].

In one of the studies, 22 MS patients were treated with NT. It was observed that NT prompted a decrease in oxidative-damage biomarker levels after 14 months and induced nuclear translocation of nuclear factor (erythroid-derived 2)-like 2 (Nrf2) which was responsible for the activation of the antioxidant pathway [55].

The level of LPS was higher in patients treated with NT compared to healthy controls in our results. Konig et al. suggested that antioxidants could reduce the level of accumulation of LPS in the mitochondria of senescent cells [59]. It might be a new target in the treatment of MS. It is possible that antioxidants added to NT therapy may help to improve its effect.

Generally, patients with RRMS had higher lipid oxidation (MDA, LHP) and TOS compared to the control group. Therefore, MS may be associated with higher peripheral OS in RRMS patients. Interestingly, similar findings were reported in other studies [23].

Tasset et al. pointed out the limitations of the studies related to peripheral samples. The authors listed such limitations as patient-specific features, the nature of the sample (tissue, cerebrospinal fluid, plasma, or erythrocytes), and differences in the study situation (experimental models and clinical studies with different characteristics). In this study, they observed an increased level of TOS, 8-OHdG, and carbonylated proteins in MS patients qualified for the treatment with NT. On the other hand, the samples were taken before administration of the drug (no information about the previous treatment) [23].

However, it can be seen that patients treated with II-line have more advanced disease than patients treated with I-line. In our results, the levels of MDA and TOS in the FG and the NT groups were not different from the control group, which may suggest a potential influence of these drugs on OS.

On the other hand, our study was limited to a small number of patients and the control group. Therefore, it was difficult to properly evaluate the effects of FG, NT, and IFN on OS in the MS patients.

The present results concerning OS level in RRMS patients in relation to gender, age, disease duration, EDSS, ARR, and Gd (+) MRI lesions are consistent with some reports of other authors.

In RRMS women treated with FG, NT, and IFN, most of the OS parameters were lowered compared to the RRMS women with the newly diagnosed disease, whereas in men only LHP was reduced in the FG group. These results are not representative enough due to the fact that the group of women was larger compared to the male group. The comparison between women and men with MS did not reveal differences (data not shown). It is known that women are more likely to have MS, which may be related to genetic predisposition [60, 61]. Most of the studies were related to the experimental sex-specific MS model, which makes it difficult to transfer these results to humans.

Mifflin et al. demonstrated that male mice with EAE given daily access to running wheels had significantly less OS compared to females with EAE. This may suggest that there are sex-specific effects on disease-related outcomes connected with exercise [62]. In contrast, Dimitrijevic et al. conducted a study on the level of OS parameters depending on the gender in rats with experimental autoimmune encephalomyelitis (EAE). In their study, in the beginning, the researchers observed an increase in xanthine oxidase (XO) activity in the spinal cord and inducible nitric oxide synthase (iNOS) mRNA expression, irrespective of the gender of the rats. Moreover, it was associated with an increase in MDA level in the spinal cord. With EAE development, superoxide dismutase (SOD) activity decreased, while O2 concentration, XO activity, and iNOS mRNA expression increased only in the spinal cord of male rats which exhibited more severe neurological symptoms compared to the female rats [63].

The data on the level of OS and gender of MS patients are insufficient to make it clear which gender predisposes to greater exposure to oxidative processes.

The level of OS in patients treated with NT and FG was not associated with new lesions on MRI. This correlation was found only for the IFN group.

In the AFFIRM study, NT was associated with a 76% reduction in new T1-hypointense lesions (the development of black holes) at 2 years (*p* < .001 versus placebo). More patients treated with NT (63%) did not present with new T1-hypointense lesions compared with those given placebo (27%). Patients treated with NT had fewer black holes, which suggested that the accumulation of axonal loss might be reduced [64, 65].

As the study showed, FG reduced brain volume loss (BVL) and promoted no evidence of disease activity (NEDA-4) in MS patients [66]. A reduction in BVL rates was observed irrespective of the levels of inflammatory lesion activity seen on MRI [67]. Our study did not evaluate BVL. It was impossible to clearly evaluate the relationship between treatment and OS on an MRI image.

No correlations between the selected OS parameters and age, disease duration, or EDSS were observed in the RRMS group treated with FG and NT.

These data are—at least in part—in agreement with other results [68]. Tasset et al. have identified oxidative/antioxidant disorders mainly expressed as GSH redox imbalance in erythrocytes in RRMS patients. Those authors also suggested that OS precedes the inflammatory response during relapse in MS patients [23]. Thus, OS parameters could become biomarkers of relapse [18]. However, it is difficult to clearly assess their impact on the EDSS scale. Our study did not allow to confirm this hypothesis because blood samples were collected at the time free from relapse.

However, one study showed an increase in OS parameters (IL-10, TNF-*α*, IFN-*γ*, advanced oxidation products, and NOx levels) along with an increase in the EDSS [52]. That study did not consider the type of treatment. Other studies conducted on patients with II-line therapy (FG, NT) did not show an increase in OS parameters such as ceramides and the EDSS scale [69]. The short-chain ceramides stimulated oxygen species production and led to neuronal death [70, 71].

Our results did not reveal EDSS differences in EDSS < 2, 2–4, and >4 groups (results not shown) and OS. In this division, however, the type of treatment was not included. No differences were observed in the whole MS group. Only patients with RRMS were included. It seems that RRMS, secondary progressive MS, and primary progressive MS patients and the control group should be compared in order to better assess the severity of the disease and OS. For example, Lam et al. observed that plasma concentrations of F2-isoprostanes and prostaglandin F2alpha (PGF2*α*) decreased and were related to the increased EDSS in patients with the progressive disease [72]. That study involved patients with the progressive disease where the processes of neurodegeneration predominated over inflammatory processes.

In our study, no correlation was found between the duration of the disease and the oxidative stress parameters of patients in I-line and II-line patients. One recent study demonstrated that NOx decreased with MS duration, which was significant for patients treated with II-line drugs [53]. This study, however, was conducted on a small group of patients.

## 6. Conclusion

In our study, patients undergoing immunomodulatory treatment presented generally lower OS parameters than the untreated patients. There is a chance that the new biomarkers may be used in the future to evaluate treatment response. The major limitation of using peripheral samples is connected with the fact that we do not know exactly how the OS parameters in serum reflect the processes occurring in the CNS during MS. Previously conducted studies were—at least in part—in agreement with our results, especially due to the fact that patients with MS have a disturbed oxidative system which results in higher OS parameters. It appears that OS parameters in serum of patients with MS did not correlate with the disease severity. It is possible that understanding the contribution of OS in MS will enable the implementation of new therapies based on the oxidative/antioxidative system. The study revealed only some differences in the oxidative system of patients treated with IFN and patients treated with II-line drugs. These differences included higher lipid peroxidation parameters in patients treated with IFN compared to the control group. Additionally, our study attempted to clarify whether II-line drugs may influence the level of OS in MS patients.

## Figures and Tables

**Table 1:** General characteristics of the study groups divided into MS patients (RRMS) and healthy people (control).

Group	RRMS	Control	*p*
*N*	121	41	0.157
Age (years)	37.5 ± 14	36 ± 21	0.588
Gender (% of females)	66.94	78.05	0.181

RRMS: relapsing-remitting multiple sclerosis; statistical significance for *p* < 0.05.

**Table 2:** The demographic and clinical characteristics of the study group.

Group	De novo RRMS	INF	FG	NT	Control
*N*	24	32	39	26	41
Age (years)	43.05 ± 12.73	40.50 ± 9.45	36.49 ± 11.67	33.96 ± 8.45	39.46 ± 12.30
Gender (% of females)	66.67	71.88	56.41	76.92	78.05
Disease duration (years)	NA	4.39 ± 4.51	6.66 ± 4.27	5.96 ± 3.35	NA
EDSS (score)	2.52 ± 1.65	1.98 ± 0.86	3.08 ± 1.10	3.08 ± 1.14	NA
ARR (*N*)	0.84 ± 0.83	0.25 ± 0.44	0.37 ± 0.64	0.12 ± 0.33	NA
Gd + MRI lesions (*N*)	0.76 ± 0.83	0.43 ± 1.13	0.09 ± 0.39	0.12 ± 0.61	NA
T2 MRI lesions (*N*)	19.05 ± 3.99	18.53 ± 4.38	20.25 ± 1.86	18.50 ± 4.90	NA

De novo RRMS: patients with a newly diagnosed relapsing-remitting multiple sclerosis; RRMS INF: RRMS patients treated with interferon beta; FG: RRMS patients treated with fingolimod; NT: RRMS patients treated with natalizumab; EDSS: Expanded Disability Status Scale; ARR: annualized relapse rate; NA: nonapplicable.

**Table:** (a) The detailed clinical characteristics of RRMS patients prior to inclusion in the II-line treatment

Group	FG	NT
Time of I-line treatment (years)	2 ± 2	2 ± 2.5
Type of treatment in I-line:		
INF beta-1a (%)	35.9	40
INF beta-1b (%)	46.15	45
OG (%)	17.95	15
EDSS (pkt)	3.5 ± 1	3.5 ± 1
ARR (*N*)	2.02 ± 0.77	2.31 ± 0.73
Gd + MRI lesions (*N*)	2 ± 4	3 ± 3
T2 MRI lesions (*N*)	18.39 ± 5.70	14.56 ± 8.19

**Table:** (b) The detailed clinical characteristics of RRMS patients after inclusion in the II-line treatment

Group	FG	NT	*p*
Time of II-line treatment (years)	2.14 ± 1.39	2.09 ± 1.27	0.841
% of patients without relapses	71.43	87.50	0.143
% of patients without clinical progression	28.57	33.33	0.721
% of patients without radiological progression	93.75	95.83	0.732
T2 MRI lesions (*N*)	20.25 ± 1.86	18.50 ± 4.90	0.380

RRMS INF: RRMS patients treated with interferon beta; FG: RRMS patients treated with fingolimod; NT: RRMS patients treated with natalizumab; OG: RRMS patients treated with octan glatiramer; EDSS: Expanded Disability Status Scale; ARR: annualized relapse rate; statistical significance for *p* < 0.05.

**Table:** (a) The comparison of the parameters of oxidative stress in serum in the study groups

Group	De novo RRMS	INF	FG	NT	Control	*p*
*N*	24	33	39	26	41	
LHP (*μ*mol/l)	24.64 ± 40.64	3.37 ± 16.32	3.07 ± 7.72	3.99 ± 7.05	0.84 ± 0.62	**0.000**
MDA (*μ*mol/l)	6.31 ± 3.18	3.11 ± 3.83	3.39 ± 1.75	3.63 ± 2.15	2.56 ± 0.51	**0.000**
TOS (*μ*mol/l)	39.11 ± 64.51	5.35 ± 35.47	8.48 ± 11.59	6.87 ± 15.4	2.39 ± 1.08	**0.000**
LPS (RF)	950.47	869.3 ± 293.9	808.52 ± 247.59	745.71 ± 260.59	764 ± 167.77	**0.021**

**Table:** (b) Post hoc analysis in the study groups

Parameter	LHP		MDA		TOS		LPS	
Group	FG	NT	FG	NT	FG	NT	FG	NT
De novo RRMS	*p* = 0.000	*p* = 0.004	*p* = 0.000	*p* = 0.000	*p* = 0.000	*p* = 0.000	NS	*p* = 0.015
INF	NS	NS	NS	NS	NS	NS	NS	NS
Control	*p* = 0.03	*p* = 0.007	NS	NS	NS	NS	NS	NS

**Table:** (c) Post hoc analysis in the study groups

Parameter	LHP		MDA		TOS		LPS	
Group	INF	Control	INF	Control	INF	Control	INF	Control
De novo RRMS	*p* = 0.000	*p* = 0.000	*p* = 0.027	*p* = 0.000	*p* = 0.003	*p* = 0.000	NS	*p* = 0.010
Control	*p* = 0.001	***x***	*P* = 0.000	*x*	*P* = 0.002	*x*	NS	*x*

De novo RRMS: patients with a new diagnosed relapsing-remitting multiple sclerosis; RRMS INF: RRMS patients treated with interferon beta; FG: RRMS patients treated with fingolimod; NT: RRMS patients treated with natalizumab; LHP: lipid hydroxyperoxides; MDA: malondialdehyde; TOS: total oxidative status; LPS: lipofuscin; NA: nonapplicable; statistical significance for *p* < 0.05.

**Table:** (a) The comparison of the selected parameters of oxidative stress in serum in women

Group	De novo RRMS	INF	FG	NT	Control	*p*
*N*	16	23	22	20	32	
LHP (*μ*mol/l)	35.65 ± 28.07	12.62 ± 16.86	8.02 ± 11.09	7.11 ± 11.04	0.86 ± 0.48	**0.000**
MDA (*μ*mol/l)	6.33 ± 2.57	4.36 ± 2.52	4.02 ± 1.94	4.10 ± 2.07	2.61 ± 0.44	**0.000**
TOS (*μ*mol/l)	21.86 ± 30.08	21.86 ± 30.08	18.04 ± 27.84	14.58 ± 17.53	2.46 ± 0.71	**0.000**

**Table:** (b) Post hoc analysis for women

Parameter	LHP		MDA		TOS	
Group	FG	NT	FG	NT	FG	NT
De novo RRMS	*p* = 0.000	*p* = 0.000	*p* = 0.000	*p* = 0.000	*p* = 0.000	*p* = 0.000
INF	NS	NS	NS	NS	NS	NS
Control	NS	NS	NS	NS	NS	NS

**Table:** (c) Post hoc analysis for women

Parameter	LHP		MDA		TOS	
Group	INF	Control	INF	Control	INF	Control
De novo RRMS	*p* = 0.000	*p* = 0.000	*p* = 0.022	*p* = 0.000	*p* = 0.001	*p* = 0.000
Control	*p* = 0.039	***x***	*P* = 0.013	*x*	NS	*x*

De novo RRMS: patients with a newly diagnosed relapsing-remitting multiple sclerosis; RRMS INF: RRMS patients treated with interferon beta; FG: RRMS patients treated with fingolimod; NT: RRMS patients treated with natalizumab; LHP: lipid hydroxyperoxides; MDA: malondialdehyde; TOS: total oxidative status; NA: nonapplicable; statistical significance for *p* < 0.05.

**Table:** (a) The comparison of the selected parameters of oxidative stress in serum in men

Group	De novo RRMS	INF	FG	NT	Control	*p*
*N*	8	9	17	6	9	
LHP (*μ*mol/l)	20.59 ± 13.87	18.57 ± 25.81	4.10 ± 3.72	9.29 ± 7.28	0.70 ± 0.51	**0.001**
MDA (*μ*mol/l)	5.73 ± 1.92	5.12 ± 3.43	3.64 ± 0.99	4.60 ± 1.79	2.43 ± 0.24	**0.003**
TOS (*μ*mol/l)	32.99 ± 21.51	31.24 ± 41.71	8.92 ± 7.05	21.48 ± 18.95	2.27 ± 0.98	**0.002**

**Table:** (b) Post hoc analysis for men

Parameter	LHP	
Group	FG	NT
De novo RRMS	*p* = 0.043	NS
INF	NS	NS
Control	NS	NS

**Table:** (c) Post hoc analysis for men

Parameter	LHP		MDA		TOS	
Group	INF	Control	INF	Control	INF	Control
De novo RRMS	NS	*p* = 0.025	NS	*p* = 0.007	NS	*p* = 0.046
Control	*p* = 0.049	*x*	*P* = 0.037	*x*	NS	*x*

De novo RRMS: patients with a newly diagnosed relapsing-remitting multiple sclerosis; RRMS INF: RRMS patients treated with interferon beta; FG: RRMS patients treated with fingolimod; NT: RRMS patients treated with natalizumab; LHP: lipid hydroxyperoxides; NA: nonapplicable; statistical significance for *p* < 0.05.

**Table 7:** The most important correlations of the selected parameters of oxidative stress in serum for RRMS patients treated with II-line drugs.

Parameter	Age (years)	Disease duration (years)	ARR	EDSS	Gd + MRI lesions (*N*)	Group
TOS	NS	NS	NS	NS	NS	FG
MDA	NS	NS	NS	NS	NS	
LHP	NS	NS	NS	NS	NS	
TOS	NS	NS	*R* = 0.479	NS	NS	NT
MDA	NS	NS	*R* = 0.412	NS	NS	
LHP	NS	NS	*R* = 0.622	NS	NS	
TOS	NS	NS	NS	NS	*R* = 0.434	IFN
MDA	NS	NS	NS	NS	*R* = 0.382	
LHP	NS	NS	NS	NS	*R* = 0.452	

FG: RRMS patients treated with fingolimod; NT: RRMS patients treated with natalizumab; EDSS: Expanded Disability Status Scale; ARR: annualized relapse rate; LHP: lipid hydroxyperoxides; MDA: malondialdehyde; TOS: total oxidative status; NA: nonapplicable; R: Pearson linear correlation coefficient.
